# Effects of orthotopic implantation of rat prostate tumour cells upon components of the *N*-acylethanolamine and monoacylglycerol signalling systems: an mRNA study

**DOI:** 10.1038/s41598-020-63198-y

**Published:** 2020-04-14

**Authors:** Mireille Alhouayek, Linda Stafberg, Jessica Karlsson, Sofia Halin Bergström, Christopher J. Fowler

**Affiliations:** 1 Department of Integrative Medical Biology, Umeå University, SE-901 87 Umeå, Sweden; 2 Present Address: Bioanalysis and Pharmacology of Bioactive Lipids Research Group, Louvain Drug Research Institute, Université catholique de Louvain, B1.72.01-1200 Bruxelles, Belgium; 3 Present Address: Apotek Hjärtat, Ringvägen 113, SE-118 60 Stockholm, Sweden; 4 Department of Medical Biosciences, Umeå University, SE-901 87 Umeå, Sweden

**Keywords:** Lipids, Prostate cancer, Cancer microenvironment, Cancer models

## Abstract

There is good evidence that the *N*-acylethanolamine (NAE)/monoacylglycerol (MAG) signalling systems are involved in the pathogenesis of cancer. However, it is not known how prostate tumours affect these systems in the surrounding non-malignant tissue and *vice versa*. In the present study we have investigated at the mRNA level 11 components of these systems (three coding for anabolic enzymes, two for NAE/MAG targets and six coding for catabolic enzymes) in rat prostate tissue following orthotopic injection of low metastatic AT1 cells and high metastatic MLL cells. The MLL tumours expressed higher levels of *Napepld*, coding for a key enzyme in NAE synthesis, and lower levels of *Naaa*, coding for the NAE hydrolytic enzyme *N*-acylethanolamine acid amide hydrolase than the AT1 tumours. mRNA levels of the components of the NAE/MAG signalling systems studied in the tissue surrounding the tumours were not overtly affected by the tumours. AT1 cells in culture expressed *Faah*, coding for the NAE hydrolytic enzyme fatty acid amide hydrolase, at much lower levels than *Naaa*. However, the ability of the intact cells to hydrolyse the NAE arachidonoylethanolamide (anandamide) was inhibited by an inhibitor of FAAH, but not of NAAA. Treatment of the AT1 cells with interleukin-6, a cytokine known to be involved in the pathogenesis of prostate cancer, did not affect the expression of the components of the NAE/MAG system studied. It is thus concluded that in the model system studied, the tumours show different expressions of mRNA coding for key the components of the NAE/MAG system compared to the host tissue, but that these changes are not accompanied by alterations in the non-malignant tissue.

## Introduction

*N*-acylethanolamines (NAEs) are a group of endogenous lipids with important biological properties. Perhaps the most well-known of these is the endogenous cannabinoid (CB) receptor ligand anandamide (arachidonoyletholamide, AEA^1^), but this group also includes the higher-abundance lipids palmitoylethanolamide (PEA), oleoylethanolamide and stearoylethanolamide, all of which all have biological activities of their own. PEA, for example, has anti-inflammatory and analgesic properties^2^, whilst oleoylethanolamide acts as a satiety agent^3^. The canonical anabolic pathway for NAEs is from the corresponding *N*-acyl-phosphatidylethanolamine (NAPE) catalyzed by the enzyme NAPE-phospholipase D (NAPE-PLD)^4,5^. NAEs are hydrolysed to their corresponding long-chain fatty acids by two enzymes, fatty acid amide hydrolase (FAAH) and *N*-acylethanolamine acid amide hydrolase (NAAA)^4,5^. However, AEA can also be oxygenated, not least by cyclooxygenase-2 to produce prostaglandin ethanolamides, which have biological actions of their own^6^. The other main endocannabinoid, 2-arachidonoylglycerol (2-AG)^7,8^, belongs to the monoacylglycerol (MAG) group of lipids. These are synthesized from the corresponding diacylglycerols (DAGs) by DAG lipases. MAGs are hydrolysed by monoacylglycerol lipase (MAGL) and the α/β-hydrolase domain containing (ABHD) 6 and 12 enzymes^4,5^, although 2-AG can also act as a substrate for FAAH^9^. As with AEA, 2-AG is a substrate for cyclooxygenase-2 to yield biologically active prostaglandin glyceryl esters^6^.

There is good evidence that the NAE and MAG systems are disturbed in human solid tumours. Thus, for example, increased levels of AEA have been found in colorectal tumour tissue^10,11^. This increase was accompanied by an increased mRNA expression and activity of both NAPE-PLD and FAAH^11^. In two large studies of patients with hepatocellular carcinoma, a high tumour MAGL expression was associated with both disease severity and a poorer survival^12,13^. In prostate cancer (PCa), both a high tumour CB_1_ receptor immunoreactivity and a high FAAH immunoreactivity are associated with disease severity and prognosis^14,15^, and NAAA is also increased^16^. In non-malignant tissue, luminal FAAH immunoreactivity shows a lower intensity of staining than the tumour FAAH immunoreactivity, and neither non-malignant CB_1_ receptor immunoreactivity nor FAAH immunoreactivity is associated with disease severity or outcome^14–17^.

**Figure 1:**
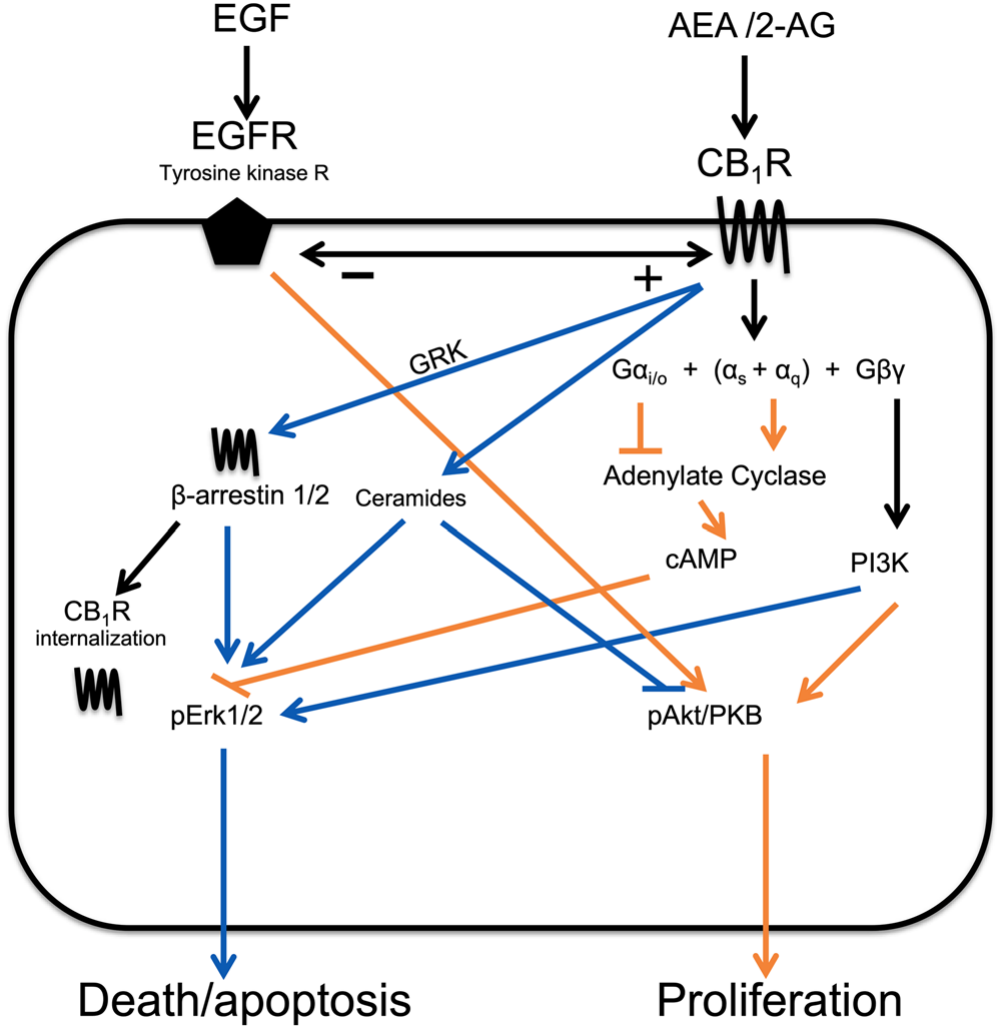
Schematic drawing of key intracellular pathways driving cell proliferation and apoptosis mediated by the interaction of AEA and 2-AG upon CB_1_ receptors. The drawing is based upon refs. ^17–33^, and in many cases synthetic- and plant-derived cannabinoids rather than endocannabinoids have been used to identify the pathways. Blue lines indicate pathways resulting in cell death/apoptosis and orange lines indicate pathways resulting in cell proliferation. → indicates stimulation, ⊥ indicates inhibition.

An important question is whether the disturbances in the NAE/MAG system in solid tumours is an unimportant biproduct of the cancer *per se*, or whether it actually contributes to cancer pathogenesis. In PCa, the association between CB_1_ receptor immunoreactivity and prognosis remains significant in a multivariate Cox regression analysis when the Gleason score (a highly predictive morphological assessment of the tumour tissue) is included in the analysis^14^. Considerable work has been undertaken with respect to signalling mediated via CB receptors in cancer cell lines and in xenograft models, where both mitogenic and anti-proliferative effects of CB receptor activation have been noted (reviews, see^18–21^). CB receptors can couple to multiple signalling pathways^22^, and the anti-proliferative effects mediated by CB receptors involve, among others, signalling via a CB_1_ receptor → phosphatidylinositol 3-kinase → extracellular signal-regulated kinase (ERK) pathway^23,24^, while mitogenic effects can be produced as a result of an activation of protein kinase B/Akt^25^. In an elegant study, Cudaback *et al*^26^ transfected astrocytoma cells with CB receptors and found that in clones with low expression levels, cannabinoids produced apoptotic effects via the ERK pathway, whereas in clones with high expression levels, the cannabinoids were mitogenic due to activation of Akt. In PCa biopsy samples, tumour CB_1_ receptor immunoreactivity and pAkt immunoreactivity are positively correlated, and cases with scores above the median for both parameters show a higher rate of cell proliferation than the other cases^27^. In addition to effects on ERK/Akt signalling, there are interactions between CB receptors and other important pathogenic signalling mechanisms, not least the epidermal growth factor (EGF) receptor pathway^28,29^. Further, changes in the activity of the intracellular signalling molecules can impact upon other systems known to be involved in the pathogenesis of cancer, such as the interleukin-6 receptor and androgen receptor pathways in the case of Pca^30–32^. Fig. 1 shows a schematic of some of the pathways involving CB receptor signalling and cell apoptosis**/**proliferation in Pca.

Given the ability of CB receptor activation to affect the fate of the tumour cells, disturbed expression of the proteins within the NAE/MAG pathways may contribute to PCa pathology in a number of ways. Thus, for example, a reduction in AEA or 2-AG levels in the tumour due to over-expression of FAAH or MAGL would be expected to be deleterious by removing an antiproliferative substrate tone mediated by the ERK pathway^17,33^, whereas an increased synthesis of these endocannabinoids could increase tumour cell proliferation in tumours with high CB_1_ receptor expression by opening up survival pathways^26^. Different coupling of surface membrane-bound and intracellular CB receptors to their effectors^34^ may also implicate different rates of receptor internalization due to different extracellular endocannabinoid concentrations. Additional CB receptor-independent pathways may also contribute: given the wide substrate specificity of FAAH and MAGL, increased expression will result in an increased production of long-chain fatty acids required to sustain tumour growth^35^.

The above discussion has focussed upon ways in which disturbed NAE/MAG signalling can contribute to the pathogenesis of cancer. Less is known about the causes for the disturbed NAE/MAG signalling systems in cancer, and whether the changes are intrinsically induced or due to external interactions where the tumours themselves affect these systems in the surrounding non-malignant tissue, termed here as “tumour instructed normal tissue” (TINT^36^). Tumour cells do not grow in isolation but influence, and are influenced by, the tumour environement^37^. One way of studying this experimentally is to follow changes in both tumour and host prostate tissue following orthotopic injection of rat R3327 (Dunning) PCa cells (originally derived from a spontaneous prostate tumour in a 22 month old Copenhagen rat, see^38^) with different metastatic abilities into the ventral prostate of syngenic Copenhagen rats^39,40^. In the present study, we have used this approach to investigate at the mRNA level the expression levels of key components of the NAE**/**MAG systems in host prostate tissue (here termed host control, HC) and the tumour tissues, and to determine whether the orthotopic injection of the PCa cells produces changes in expression of these components in TINT.

## Results

### Orthotopic injection experiments: host control (HC) tissue

**Figure 2:**
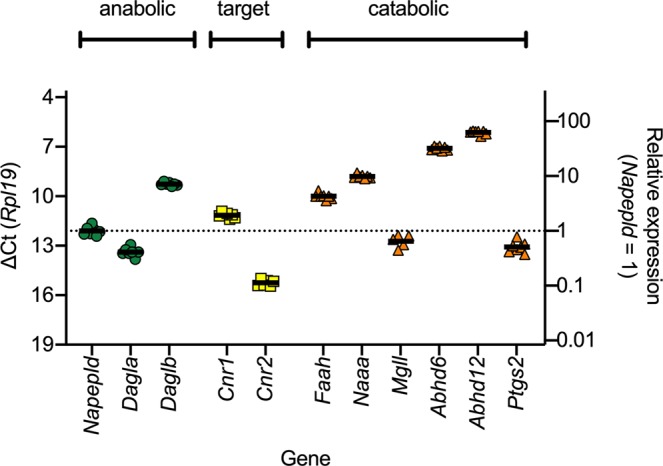
mRNA levels of genes coding for components of the NAE/MAG signalling pathways in host control prostate tissue. Shown are individual values with solid lines representing the mean ∆Ct values with *Rpl19* as the reference gene. The left axis is reversed so that a higher expression of mRNA is upwards. The right axes show the antilogged geometric means for the genes expressed relative to the antilogged geometric mean for *Napepld*.

In the orthotopic injection experiments, either vehicle or PCa cells (poorly metastatic Dunning AT1 and highly metastatic Dunning MatLyLu [MLL] cells) were injected orthotopically into the ventral prostate of syngenic Copenhagen rats and tissue was collected 10 days later (see^40^ and Methods). At this time point, the tumours occupy about 30% of the prostate lobe, but have not yet metastasised to the lymph node to any detectable extent^40^. In Fig. 2, the qPCR data quantifying mRNA levels for eleven genes coding for components of the NAE and MAG systems are shown for the host control prostate tissue. The primer pairs used are given in Supplementary Table S1. The data are presented as ∆Ct values with *Rpl19* as reference gene to show expression levels. A difference of +1 and −1 between two mean ∆Ct values corresponds to an absolute difference of 0.5 and 2, respectively, for the geometric means of the data expressed as 2^-∆∆Ct^. Such values relative to *Napepld* expression (chosen simply because it has intermediate expression levels compared with the other genes) are shown on the right y-axis. With respect to genes coding for 2-AG anabolic enzymes, *Daglb* levels are an order of magnitude higher than *Dagla* levels. For the genes coding for the CB receptors (i.e. the targets of AEA and 2-AG, but not the other NAEs and MAGs), *Cnr1* levels are an order of magnitude higher than *Cnr2* levels. For the genes coding for NAE hydrolytic enzymes, *Faah* and *Naaa* have rather similar levels. In contrast, for the genes coding for MAG hydrolytic enzymes, levels of *Mgll* are 1–2 orders of magnitude lower than *Abhd6* and *Abhd12*.

### Orthotopic injection experiments: Tumour tissue

**Figure 3:**
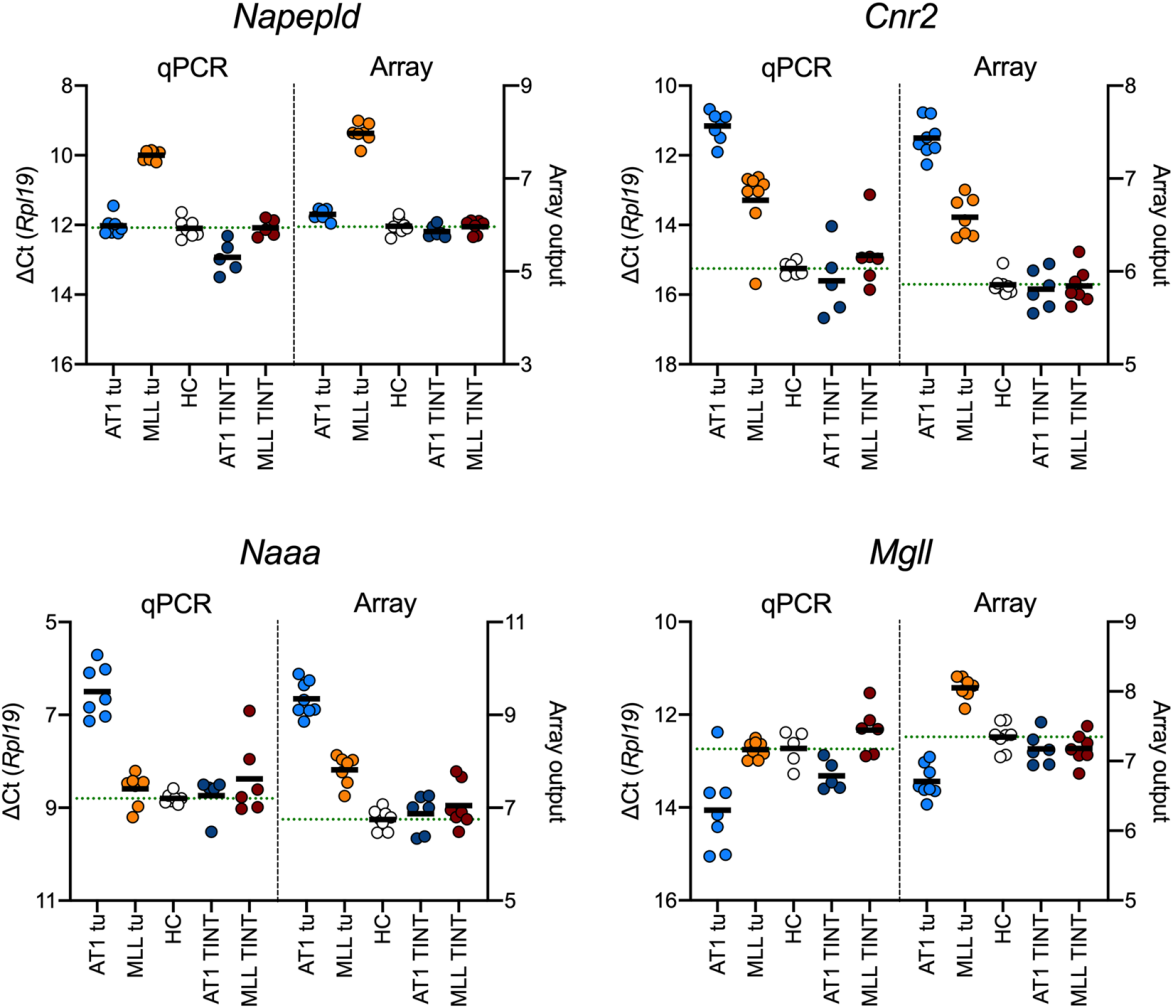
Scatterplots of *Napepld*, *Cnr2*, *Naaa* and *Mgll* gene expression in host control (HC), tumour (tu) tissue and TINT. Comparison of qPCR and Array data. Left axes show the ∆Ct from the qPCR experiments with *Rpl19* as reference gene. The right axes show the array data, as normalised values on a log_2_ scale, taken from S1 Dataset in Strömvall *et al*^40^. The statistical analyses of these data are presented in Tables 1 and 2 (qPCR) and Supplementary Tables S2 and S3 (array). Note that the left y-axis but not the right y-axis has been reversed so that for both qPCR and Arrays, the direction of change (−1 = doubling for qPCR, +1 = doubling for array) is the same.

The qPCR data for the tumour tissue are compared with the host control data in Table 1, with examples of the scatterplots shown in Fig. 3 for *Napepld*, *Cnr2*, *Naaa* and *Mgll*. The scatterplots for the other genes are shown in Supplementary Fig. S1. The tissue samples were originally prepared for a gene microarray study^40^, and for comparative purposes, the corresponding array values extracted from the database in that study are shown in Fig. 3 and in Supplementary Table S2. In the qPCR experiments, we quantified mRNA for three reference genes, *Rpl19*, *Rps12* and *Psmc4*. This means that there are seven combinations of reference genes that can be used for the statistical analysis. We have presented the data with *Rpl19* alone as reference gene, but also run ANOVAs for the other six combinations and shown the range of P values found in Table 1 (shown as “min others” and “max others”). For example, the ANOVA P value for *Napepld* with *Rpl19* as reference gene was 1 × 10^−9^, whilst for the other six combinations the range was <1 × 10^−9^ to 2 × 10^−9^, suggesting a highly robust effect. In contrast the P value for *Ptgs2* (coding for cyclooxygenase-2) with *Rpl19* as reference gene was 0.012, but the P values for the six other combinations ranged from 5.3×10^−4^ to 0.2. Additionally, for the ∆Ct values with *Rpl19* as reference gene we implemented a 5% false discovery rate for the ANOVA P values.

**Table 1:** Comparison of the mRNA ∆Ct values for host control (HC), AT1 tumour tissue and MLL tumour tissue using *Rpl19* as reference gene.

	Mean	SD	N	Comparison:	ANOVA P	post-hoc Dunnett’s T3 tests		
					(Welch)	HC vs AT1	HC vs MLL	AT1 vs MLL
***Genes coding for anabolic enzymes***								
*Napepld*								
Host control	12.10	0.27	7	∆Ct (*Rpl19*):	1 × 10^−9^	0.94	6.1 × 10^−8^	1.1 × 10^−7^
AT1 tumour	12.03	0.28	7	min others:	<1 × 10^−9^	0.0046	9.0 × 10^−9^	3.1 × 10^−7^
MLL tumour	10.00	0.14	7	max others:	2.0 × 10^−9^	0.87	8.8 × 10^−8^	4.5 × 10^−6^
*Dagla*								
Host control	13.38	0.27	7	∆Ct (*Rpl19*):	1.2 × 10^−8^	1.6 × 10^−8^	0.0013	0.31
AT1 tumour	10.60	0.37	7	min others:	8.0 × 10^−9^	1.3 × 10^−8^	7.8 × 10^−4^	0.44
MLL tumour	11.25	1.02	8	max others:	1.7 × 10^−7^	4.5 × 10^−7^	0.0018	0.99
*Daglb*								
Host control	9.26	0.10	7	∆Ct (*Rpl19*):	3.5 × 10^−7^	9.1 × 10^−7^	0.99	1.3 × 10^−5^
AT1 tumour	8.59	0.13	7	min others:	1.4 × 10^−7^	2.7 × 10^−7^	1.6 × 10^−5^	1.2 × 10^−5^
MLL tumour	9.29	0.21	8	max others:	0.024	0.028	0.77	0.38
***Genes coding for CB receptors***								
*Cnr1*								
Host control	11.15	0.16	7	∆Ct (*Rpl19*):	2.7 × 10^−4^	4.8 × 10^−4^	0.044	0.96
AT1 tumour	15.19	1.28	7	min others:	1.3 × 10^−4^	2.2 × 10^−4^	0.040	0.95
MLL tumour	15.74	2.63	5	max others:	3.3 × 10^−4^	5.3 × 10^−4^	0.053	>0.99
*Cnr2*								
Host control	15.25	0.19	6	∆Ct (*Rpl19*):	1 × 10^−9^	8.0 × 10^−9^	0.0021	9.1 × 10^−4^
AT1 tumour	11.16	0.43	7	min others:	1 × 10^−9^	1.1 × 10^−8^	0.0012	0.0031
MLL tumour	13.29	1.02	8	max others:	7.0 × 10^−9^	1.4 × 10^−7^	0.0029	0.018
***Genes coding for catabolic enzymes***								
*Faah*								
Host control	10.00	0.18	7	∆Ct (*Rpl19*):	5.9 × 10^−6^	2.5 × 10^−4^	7.6 × 10^−4^	0.91
AT1 tumour	13.62	1.01	7	min others:	1.6 × 10^−6^	3.2 × 10^−5^	5.4 × 10^−4^	0.32
MLL tumour	13.26	1.35	8	max others:	7.1 × 10^−6^	2.3 × 10^−4^	0.0012	0.85
*Naaa*								
Host control	8.80	0.12	7	∆Ct (*Rpl19*):	4.5 × 10^−6^	3.8 × 10^−5^	0.32	3.2 × 10^−5^
AT1 tumour	6.50	0.55	7	min others:	5.7 × 10^−8^	3.3 × 10^−6^	3.7 × 10^−4^	4.0 × 10^−6^
MLL tumour	8.59	0.33	8	max others:	8.8 × 10^−6^	1.2 × 10^−4^	0.14	1.0 × 10^−4^
*Mgll*								
Host control	12.73	0.38	5	∆Ct (*Rpl19*):	0.025	0.026	>0.99	0.028
AT1 tumour	14.06	0.93	7	min others:	0.0059	0.012	0.014	0.0074
MLL tumour	12.75	0.18	8	max others:	0.019	0.37	0.98	0.044
*Abhd6*								
Host control	7.10	0.12	7	∆Ct (*Rpl19*):	1.5 × 10^−7^	1.5 × 10^−6^	4.9 × 10^−5^	6.9 × 10^−3^
AT1 tumour	8.09	0.20	7	min others:	5.7 × 10^−8^	1.1 × 10^−6^	1.2 × 10^−5^	4.2 × 10^−4^
MLL tumour	9.00	0.58	8	max others:	1.4 × 10^−5^	0.034	3.3 × 10^−5^	0.18
*Abhd12*								
Host control	6.14	0.11	7	∆Ct (*Rpl19*):	2.2 × 10^−5^	3.5 × 10^−4^	0.0017	0.041
AT1 tumour	6.89	0.27	7	min others:	1.9 × 10^−6^	1.6 × 10^−5^	6.4 × 10^−4^	0.013
MLL tumour	7.78	0.78	8	max others:	0.0045	0.73	0.0056	0.45
*Ptgs2*								
Host control	13.08	0.35	7	∆Ct (*Rpl19*):	0.012	0.011	>0.99	0.18
AT1 tumour	12.25	0.49	7	min others:	5.3 × 10^−4^	5.3 × 10^−4^	0.42	0.25
MLL tumour	13.14	1.13	8	max others:	0.20	0.20	>0.99	0.83
***Other genes***								
*Tnfa*								
Host control	13.13	0.60	7	∆Ct (*Rpl19*):	1.1 × 10^−5^	2.0 × 10^−5^	>0.99	1.5 × 10^−4^
AT1 tumour	10.74	0.59	7	min others:	2.5 × 10^−6^	2.5 × 10^−6^	0.22	2.0 × 10^−4^
MLL tumour	13.10	0.88	8	max others:	1.0 × 10^−4^	1.5 × 10^−4^	0.99	0.0016

For the ANOVA P values for ∆Ct using *Rpl19* as reference gene, which were calculated not assuming equal SD values, the critical value of P assuming a 5% false discovery rate^72^ was 0.05. “min others” and “max others” show the range of P values for the other combinations of reference genes.

**Figure 4:**
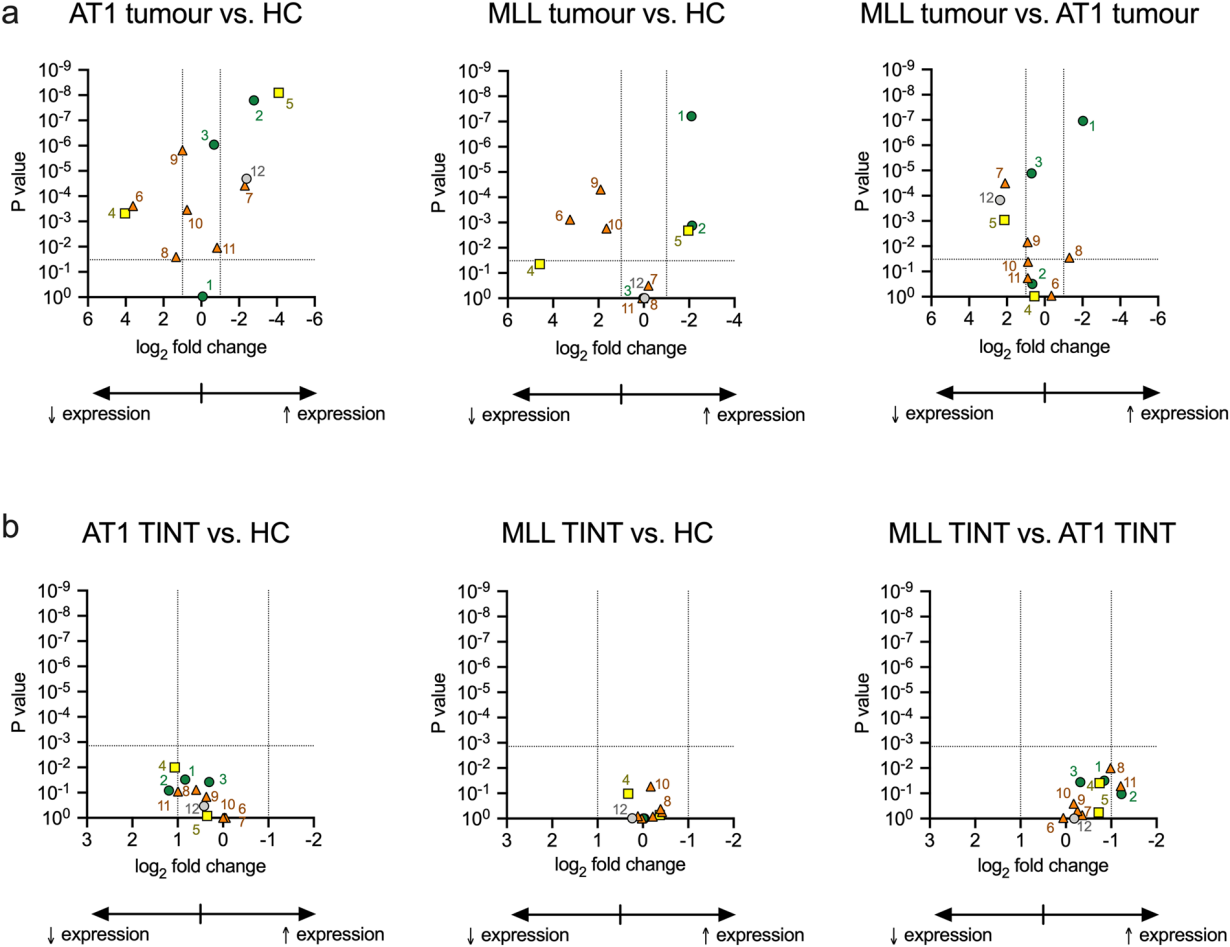
Volcano plots of bivariate comparisons between a, host control and tumour tissue; b, host control and TINT. The log_2_ fold change is calculated from the mean ∆Ct (*Rpl19* as reference gene) values summarized in Tables 1 and 2. Note the different scales in Panels a and b. The P values are for the post-hoc comparisons given in these Tables. The vertical dotted lines show a fold change of ±1, i.e. a halving/doubling of mRNA expression. The horizontal lines show the critical value of P assuming a 5% false discovery rate (0.033 for Panel a, 0.0014 for Panel b). The genes are numbered as follows: 1, *Napepld*; 2, *Dagla*; 3, *Daglb*; 4, *Cnr1*; 5, *Cnr2*; 6, *Faah*; 7, *Naaa*; 8, *Mgll*; 9, *Abhd6*; 10, *Abhd12*; 11, *Ptgs2* and 12, *Tnfa*.

The data in Table 1 is somewhat indigestible, but the results can be visualised in Volcano plots^41^, where the x axis shows the change in expression and the y axis shows the P values. Genes outside the boundary lines (a doubling**/**halving of gene expression and the critical value of P assuming a 5% false discovery rate) were deemed to be of potential interest. The Volcano plots for the bivariate comparisons are shown in Fig. 4a. The corresponding Volcano plots for the array data are shown in Supplementary Fig. S2a. With respect to the genes coding for the anabolic enzymes, *Napepld* levels were higher in the MLL tumour than either the AT1 tumour or HC tissue, while *Dagla* is higher in the tumour tissues than in the HC tissue. For the targets for AEA and 2-AG, *Cnr1* levels were lower and *Cnr2* levels higher in the tumour tissue than in the HC tissue, and the *Cnr2* levels higher in the AT1 tumour than the MLL tumour. For the genes coding for NAE hydrolysis, *Faah* levels were lower in the tumour tissue than in the HC, whilst *Naaa* levels were higher in the AT1 tumour tissue than the other samples. Finally, for the genes (other than *Faah*) coding for MAG hydrolysis there were no dramatic differences between the samples, with the possible exception of *Mgll*, which was lower in the AT1 tumours than the other samples.

### Orthotopic injection experiments: TINT

The qPCR data for the TINT samples (with the same HC samples) are summarised in Table 2, with examples in Fig. 3 and Volcano plots shown in Fig. 4b. The corresponding array data are summarised in Supplementary Table S3 and in Supplementary Fig. S2b. In contrast to the dramatic differences in gene expression between the tumour tissue samples and HC, there were no obvious changes in gene expression seen in the TINT samples. This would suggest that pathogenic factors released by the tumours do not influence expression of genes coding for components of the NAE/MAG system in this rat orthotopic model.

**Table 2:** Comparison of the mRNA ∆Ct values for host control (HC), AT1 TINT tissue and MLL TINT tissue using *Rpl19* as reference gene.

	Mean	SD	N	Comparison:	ANOVA P	post-hoc Dunnett’s T3 tests		
					(Welch)	HC vs AT1	HC vs MLL	AT1 vs MLL
***Genes coding for anabolic enzymes***								
*Napepld*								
Host control	12.10	0.27	7	∆Ct (*Rpl19*):	0.019	0.031	>0.99	0.031
AT1 TINT	12.93	0.46	5	min others:	0.0064	0.019	0.51	0.014
MLL TINT	12.09	0.25	5	max others:	0.015	0.026	>0.99	0.024
*Dagla*								
Host control	13.38	0.27	7	∆Ct (*Rpl19*):	0.071	0.082	>0.99	0.11
AT1 TINT	14.58	0.88	5	min others:	0.054	0.079	0.92	0.058
MLL TINT	13.35	0.76	6	max others:	0.072	0.10	>0.99	0.11
*Daglb*								
Host control	9.26	0.10	7	∆Ct (*Rpl19*):	0.024	0.038	>0.99	0.036
AT1 TINT	9.57	0.18	5	min others:	0.0024	0.021	0.20	0.0036
MLL TINT	9.25	0.13	6	max others:	0.029	0.037	0.97	0.076
***Genes coding for CB receptors***								
*Cnr1*								
Host control	11.15	0.16	7	∆Ct (*Rpl19*):	0.0033	0.010	0.10	0.039
AT1 TINT	12.22	0.45	5	min others:	4.8 × 10^−4^	8.5 × 10^−4^	0.14	0.0029
MLL TINT	11.48	0.28	6	max others:	0.0016	0.0055	0.66	0.032
*Cnr2*								
Host control	15.25	0.19	6	∆Ct (*Rpl19*):	0.54	0.84	0.72	0.57
AT1 TINT	15.60	1.04	5	min others:	0.38	0.78	0.55	0.40
MLL TINT	14.88	0.93	6	max others:	0.54	0.82	0.77	0.58
***Genes coding for catabolic enzymes***								
*Faah*								
Host control	10.00	0.18	7	∆Ct (*Rpl19*):	0.85	0.97	>0.99	0.94
AT1 TINT	9.97	0.09	5	min others:	0.14	0.88	0.31	0.14
MLL TINT	10.02	0.24	6	max others:	0.98	1.00	>0.99	1.00
*Naaa*								
Host control	8.80	0.12	7	∆Ct (*Rpl19*):	0.51	0.98	0.55	0.73
AT1 TINT	8.74	0.44	5	min others:	0.32	>0.99	0.33	0.37
MLL TINT	8.38	0.81	6	max others:	0.54	>0.99	0.58	0.70
*Mgll*								
Host control	12.73	0.38	5	∆Ct (*Rpl19*):	0.011	0.077	0.42	0.010
AT1 TINT	13.32	0.32	5	min others:	0.0011	0.014	0.15	0.0027
MLL TINT	12.33	0.50	6	max others:	0.0069	0.057	0.58	0.0097
*Abhd6*								
Host control	7.10	0.12	7	∆Ct (*Rpl19*):	0.13	0.15	0.80	0.50
AT1 TINT	7.46	0.32	5	min others:	0.068	0.096	0.69	0.080
MLL TINT	7.21	0.29	5	max others:	0.10	0.12	0.99	0.69
*Abhd12*								
Host control	6.14	0.11	7	∆Ct (*Rpl19*):	0.062	>0.99	0.053	0.26
AT1 TINT	6.13	0.18	5	min others:	0.0016	0.76	0.0070	0.0032
MLL TINT	5.96	0.11	6	max others:	0.34	0.97	0.75	0.41
*Ptgs2*								
Host control	13.08	0.35	7	∆Ct (*Rpl19*):	0.050	0.090	0.83	0.052
AT1 TINT	14.07	0.72	5	min others:	0.013	0.033	0.45	0.011
MLL TINT	12.86	0.63	6	max others:	0.033	0.065	0.93	0.038
***Other genes***								
*Tnfa*								
Host control	13.13	0.60	7	∆Ct (*Rpl19*):	0.34	0.34	0.98	0.99
AT1 TINT	13.55	0.28	5	min others:	0.30	0.31	0.94	0.89
MLL TINT	13.37	1.51	6	max others:	0.35	0.35	>0.99	0.99

Note that the HC values are the same as in Table 1. For the ANOVA P values, calculated not assuming equal SD values, the critical value of P assuming a 5% false discovery rate^72^ was 0.0042. “min others” and “max others” show the range of P values for the other combinations of reference genes.

#### AT1 cells in culture: comparison with HC rat prostate

**Figure 5:**
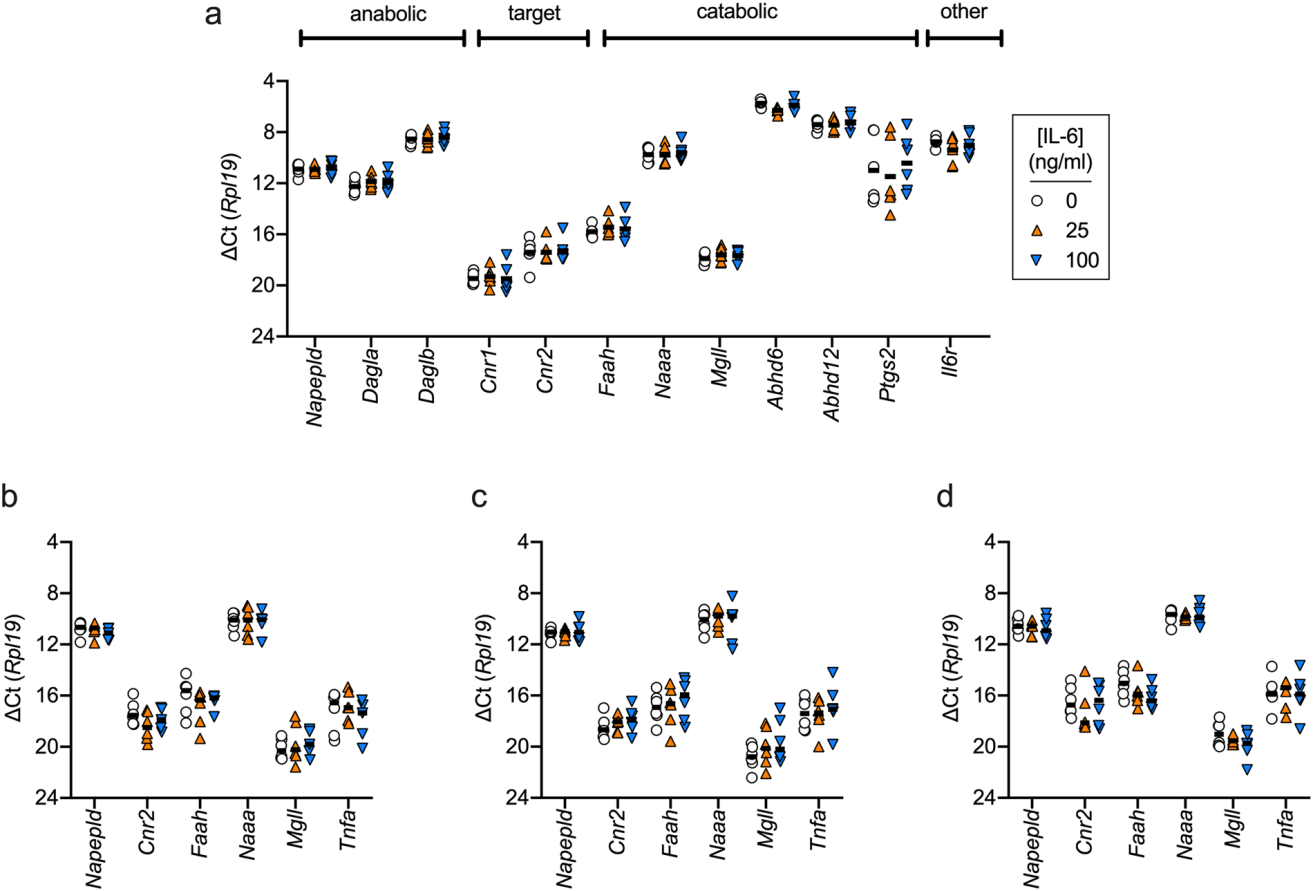
Effect of IL-6 treatment of AT-1 cells upon the mRNA expression levels of genes coding for components of the NAE/MAG system. AT1 cells were treated for either 3 h (Panels a-c) or 24 h (Panel d) with the concentrations of IL-6 shown prior to determination of mRNA levels. The treatment conditions were serum-free Krebs-Ringer buffer (Panel a), serum-free medium (Panel b) and medium containing 1% FBS (Panels c and d). Individual values are shown (N = 5-6) with solid lines representing the mean ∆Ct values with *Rpl19* as the reference gene. ANOVA P values for mixed effects models (REML) not assuming sphericity were determined for each of the genes. In all cases except for *Abhd6* in panel A, the P values for the effect of treatment were not significant. For *Abhd6*, the P value was 0.014, and Tukey’s multiple comparisons test gave significant differences between the mean for 25 ng/ml IL-6 and the other two treatments. However, the P value does not take into account multiple comparisons (there are 29 P values for the data in the figure), and the critical value of P assuming a 5% false discovery rate was 0.0017.

Experiments were also conducted using AT1 cells in culture. For the control cells (from the experiments detailed below, see Fig. 5), the relative expression of genes coding for components of the NAE**/**MAG system was broadly similar to that of the HC rat prostate (i.e. *Daglb* > *Dagla*; *Abhd6* and *Abhd12* > *Mgll*). There were, however, two exceptions: in the AT1 cells, *Cnr1* expression was lower, and similar to *Cnr2* expression, and *Faah* expression was much lower than *Naaa* expression (see Supplementary Fig. S3 for a comparison between AT1 cells and HC tissue). For the AT1 cells, the mean (±SD, N = 6) ∆Ct for *Faah* and *Naah* were 15.80 ± 0.41 and 9.76 ± 0.58. The difference in mean values (−6.03) corresponds to a relative expression of *Faah*:*Naaa* of 1:65.

### AT1 cells in culture: hydrolysis of [^3^H]AEA

The low expression of *Faah* compared to *Naaa* in the AT1 cells raises the possibility that in these cells, NAAA rather than FAAH is primarily responsible for the hydrolysis of AEA. In order to investigate this possibility, the hydrolysis of 0.1 µM [^3^H]AEA was investigated. The cells were preincubated with either an inhibitor of FAAH (URB597^42^, 1 µM) or NAAA (pentadecylamine^43^, 30 μM), or both together prior to addition of [^3^H]AEA and incubation for a further 15 min. This was undertaken both for cells cultured in serum-free medium and in medium containing 0.1% foetal bovine serum (FBS) prior to the experiment. The data are shown in Table 3, where a significant effect of URB597, but not of pentadecylamine or of the interaction URB597 x pentadecylamine was found. Expressing the data as % of the corresponding vehicle control, the values for serum-free and 1% FBS samples, respectively, were: URB597, 19 ± 4 (15–24) and 21 ± 8 (13–29); pentadecylamine, 94 ± 20 (73–115) and 111 ± 13 (97–125); URB597 + pentadecylamine, 13 ± 2 (9–18) and 10 ± 3 (3–18) (means ± SD, with 95% confidence limits in the brackets). Thus, under the conditions used, the majority of the exogenously supplied AEA is hydrolysed by FAAH rather than by NAAA in the intact AT1 cells.

**Table 3:** Hydrolysis of [^3^H]AEA by intact AT1 cells.

Treatment	[^3^H]AEA hydrolysis (pmol/mg protein)			
	Serum-free		1% FBS	
	Mean	SD	Mean	SD
Vehicle	15.4	8.0	14.7	7.4
URB597 (1 µM)	3.0	1.6	3.1	1.9
Pentadecylamine (30 µM)	14.0	6.8	16.4	8.5
URB597 + Pentadecylamine	1.9	0.75	1.5	1.0

AT1 cells were incubated for 3 h with either serum-free or serum containing (1% FBS) medium and then washed with Krebs-Ringer HEPES bicarbonate buffer prior to preincubation for 10 min at 37 °C with either vehicle or the compounds shown. Thereafter, [^3^H]AEA (diluted with non-radioactive AEA to give an assay concentration of 100 nM) was added and the samples were incubated for a further 15 min with the radioactive compounds prior to assay work-up (see Methods for details). Shown are means ± SD, N = 6 and expressed as pmol per mg protein, where protein contents were determined in parallel wells. A repeated measures ANOVA with heteroscedascity hc3 correction and with matching for the experimental day gave a significant main effect of URB597 (P = 0.0047), whereas neither the main effect of pentadecylamine, the main effect of serum, nor any of the interactions were significant, defined as P < 0.05.

### AT1 cells in culture: effect of interleukin-6 (IL-6) treatment

The Volcano plots for the tumours compared to the HC cells (Fig. 4a) indicate considerable differences in relative expression levels for several of the components of the NAE/MAG systems. As pointed out in the discussion, such changes may be the result of the tumourigenesis per se, but could also be due to the influence of factors emanating from the tumour environment. One potential influence could be that of inflammatory cytokines, given that in the orthotopic model used here, extra-tumoural macrophages increase the growth of tumours, while in turn the tumours differentially affect genes in the host tissue associated with inflammatory responses^39,40^. There is data in both PCa cells and other cells that several of the components of the NAE system (NAE levels, expression of NAPE-PLD, CB receptors, FAAH) can be modulated by inflammatory stimuli^44–51^, (and vice versa, see e.g^52–56^.). One inflammatory cytokine that has not been studied in PCa cells in this respect is IL-6. This is a notable omission, given that this cytokine is involved in the pathogenesis of PCa^57^, can be released from PCa cells in response to treatment with a metabolically stable analogue of AEA^58^, and has been reported to affect CB receptor mRNA expression in whole blood^59^.

Here, AT1 cells were treated either with vehicle or IL-6 (25 and 100 ng/ml) for 3 or 24 h. The initial studies were performed where the cells in serum-free Krebs-Ringer buffer were exposed to IL-6 for 3 h (Fig. 5a). We included *Il6r* in those experiments to confirm that the target for IL-6 was expressed, at the mRNA level, in the AT1 cells. Subsequent experiments investigated *Napepld*, *Cnr2*, *Faah*, *Naaa*, *Mgll* and *Tnfa* (included in view of the known effects of TNFα upon components of the NAE/MAG systems in PCa cells^50^) for cells in serum-free medium treated with IL-6 for 3 h (Fig. 5b) or in serum-containing medium treated for 3 h (Fig. 5c) or 24 h (Fig. 5d).

As is clear from the data in Fig. 5, no robust effects of IL-6 were seen under the conditions used. The only significant effect was upon *Abhd6* (Fig. 5a). However, the P value (0.014) was well outside the critical value of 0.0017 for all 29 P values obtained in these experiments. Furthermore, the difference in mean ∆Ct values between the 25 ng/ml IL-6-treated and either control or 100 ng/ml IL-6-treated cells was 0.55 and 0.40, respectively [corresponding to a 32% and 24% decrease, respectively], and thus would be well inside the ±1 fold change cut-off lines if the data were presented as a Volcano plot.

## Discussion

The present study was designed to explore at the mRNA level the difference in expression of key components of the NAE and MAG systems in PCa tumours and host tissue, and to determine whether or not the tumour growth was accompanied by changes in these components in the TINT. The main findings of the study are discussed below:

### In the rat prostate (HC) samples, *Cnr1* levels are greater than *Cnr2* levels, while *Mgll* levels are lower than either *Abhd6* or *Abhd12* levels

With respect to *Cnr1* and *Cnr2*, the data are consistent with the human prostate, where detectable levels of *CNR1* but not *CNR2* have been reported^59^. At the protein level in humans, CB_1_ receptor immunoreactivity is found at the plasma membrane of both basal and luminal epithelial cells, and with little or no staining of the stroma^14,60^. These receptors are functional, since incubation of human prostate membranes with CB_1_ receptor agonists produces a decreased adenylyl cyclase activity in a pertussis toxin-sensitive manner^60^. A similar immunochemical location of CB_1_ receptors is seen in the rat, and in isolated organ bath experiments, stimulation of these receptors inhibits the contraction of the prostate gland^61^.

With respect to *Mgll*, *Abhd6* and *Abhd12*, the results are at first sight surprising, since MAGL is often characterized as the primary hydrolytic enzyme for 2-AG. To our knowledge, the relative expression of these hydrolytic enzymes in human prostate tissue has not been explored, but in DU145 and PC3 cells, *MGLL*, *ABHD6* and *ABHD12* expression is roughly equal^62^. The pattern seen in the present study is similar to that seen in human SHSY5Y neuroblastoma cells, and in these cells, inhibition of ABHD6 but not MAGL *per se* reduces their ability to hydrolyse exogenously added 2-oleoylglycerol. In contrast, in the DU145 cells, the reverse pattern is seen^62^.

### The expression of the components of the NAE/MAG systems are very different in the tumours than in the HC tissue

The tumour tissue shows increased *Dagla* and *Cnr2* levels and reduced *Cnr1* and *Faah* levels compared to the HC tissue; the AT1 tumours show increased *Naaa* levels and the MLL tumours show increased *Napepld* levels. Interpretation of these data is far from simple given that the tumours (and the HC) are heterogeneous in nature and that some of the changes (such as for *Cnr2*^59^ and *Naaa*^63^) may reflect the presence of immune cells in the tumour. It can be noted, however, that the expression of *Cnr1* and *Faah* is very low in the AT1 cells, and so this at least matches the case for the AT1 tumours (Fig. 4A). Perhaps the most interesting finding is the pattern of an increased *Napepld* expression in the highly metastatic MLL tumours compared to the low metastatic AT1 tumours. Assuming this change is also seen at the protein level (such as is found in colorectal cancer^11^) the data suggest that the more malignant tumours have a higher NAE synthetic capacity. However, it is wise to exercise caution in interpreting these data, but at the very least they motivate follow-up studies using *in situ* hybridization and immunochemical techniques as well as measurements of NAE levels in the tumours and TINT.

The different expression of components of the NAE**/**MAG systems in the tumours and HC tissue is reminiscent of the situation in human PCa. Most work has looked at cultured cells, where for example differences in the expression of both *NAAA* and *FAAH* is higher in androgen-sensitive LNCaP cells than in the androgen-resistant DU145 and PC3 cells or in cultured prostate epithelial cells (PrEC)^17,64^. A higher FAAH immunoreactivity is seen in PCa tumour tissue than in the luminal cells in the TINT tissue^15,17^. In addition to intrinsic changes associated with tumourigenesis, environmental factors emanating from the tumour environment may be associated with changes in expression of components of the NAE**/**MAG systems. Obvious candidates are cytokines, given that inflammatory changes are seen in PCa tissue^65^ and in the orthotopic model used here^39,40^. Relatively little work has been undertaken upon the effects of cytokines upon expression of the components of the NAE**/**MAG systems in PCa cells. To our knowledge, the only published studies have concerned IL-4, which increases the activity of FAAH in AT1 cells and in PC3 cells (consistent with effects in human lymphocytes^44^), while TNFα increases *PTGS2* and decreases *NAPEPLD*, *DAGLa* and *DAGLb* mRNA levels in human androgen-resistant DU145 cells^15,50^. Thus, the data with IL-6, albeit negative, increase our knowledge in this area. Clearly, more work is needed to delineate the interplay between cytokines and the NAE**/**MAG systems in PCa.

The low expression of *Faah* compared with *Naaa* in the AT1 cells has been investigated in more detail. The substrate specificities of the two enzymes differ somewhat, whereby FAAH hydrolyses AEA more readily than PEA, whereas the reverse is true for NAAA^63,66^. The large difference in expression levels in the AT1 cells, raises the possibility that NAAA is the primary catabolic enzyme for AEA in these cells. However, with respect to the ability of the intact cells to hydrolyse extracellularly administered AEA, no measureable component of the hydrolysis due to NAAA was seen, indicating that FAAH is the primary hydrolytic enzyme for AEA even when expression levels are much smaller than NAAA. At the mRNA level, expression of *NAAA* is higher in the prostate than in other tissues^67^, and a glycoproteomic analysis of prostate tissues (10 normal, 24 non-aggressive tumours, 16 aggressive tumours and 25 metastatic tumours) indicated that NAAA levels are associated with tumour aggressivity^16^. The authors suggested that this association may be related to its ability to hydrolyse PEA, which has anti-inflammatory properties via mechanisms unrelated to CB receptors^68^, to its corresponding long-chain fatty acid. Our data argues that this suggestion is more likely than to an effect of an increased NAAA expression upon AEA hydrolysis and hence CB receptor signalling.

### The expression of the components of the NAE/MAG systems in the TINT is not overtly changed

Albeit a negative finding, this is an interesting observation since it demonstrates that the tumour growth (and all the factors secreted by the tumours to promote its growth) does not trigger changes in the key components of the NAE**/**MAG system in the TINT, at least at the level of mRNA. This is in contrast to changes in expression of genes such as *Hmox1* (coding for haemoxygenase-1, upregulated) and *Mmp7* (coding for matrix metallopeptidase 7, downregulated) that are seen in the TINT in the orthotopic model^40^. Indeed, in that study, not only changes vs. HC tissue were seen, but also between the TINTs for the AT1 and MLL cells, not least with respect to genes coding for proteins involved in suppression of anti-immune responses of the host tissue^40^. The lack of change for *Faah* and *Cnr1* in the TINT seen here is reminiscent of the situation in PCa patients, where tumour, but not TINT FAAH and CB_1_ receptor immunoreactivity is associated with disease severity and outcome^14,15^.

In conclusion, the present pilot study has indicated that the inflammatory changes found in TINT following orthotopic injection into Copenhagen rats of syngenic tumour cells^39,40^ is not accompanied by overt changes at the mRNA level of components of the NAE**/**MAG system, whereas the tumours show different expression patterns to the HC. These studies motivate further *in situ* hybridization, immunochemical staining, enzyme activity measurement and NAE quantification studies for both the orthotopic and cell culture approaches to establish whether these different expression patterns reflect a changed ability of the tumour cells to produce and catabolise NAE and MAGs.

## Methods

### Materials

Tritium labelled AEA (ethanolamide-1-^3^H, [^3^H]-AEA, specific activity 60 Ci/mmol) was purchased from American Radiolabeled Chemicals, Inc (St Louis, MO, USA). Unlabelled AEA, arachidonic acid and URB597 (cyclohexylcarbamic acid 3′-carbamoyl-biphenyl-3-yl ester) were purchased from the Cayman Chemical Co. (Ann Arbor, MI, USA). Dynabeads mRNA DIRECT Purification Kit, *L*-glutamine, Trypsin-EDTA and trypan blue, penicillin and streptomycin, and Pierce BCA Protein Assay Kit were bought from ThermoFisher Scientific (Waltham, USA). RPMI 1640 cell culture medium was bought from Gibco by Life Technologies. Recombinant rat IL-6 (in phosphate-buffered saline (PBS) supplemented with 0.1% bovine serum albumin (BSA)) was obtained from R&D systems (Abingdon, UK). Dexamethasone and pentadecylamine were obtained from Sigma Aldrich (St Louis, MO, USA). For the qPCR experiments, primers (Supplementary Table S1) were bought from Integrated DNA Technologies (Leuven, Belgium). 2x SYBR green-separate ROX mix was bought from qPCR Biosystems (London, UK). Water was purified by a Milli-Q Gradient system (Millipore, Milford, MA, USA).

### Orthotopic injection of AT1 and MLL cells

The animal experiments were approved by the Umeå Ethical Committee for animal research (permit number A 42-15A). The experiments, which were performed in accordance with Swedish guidelines and regulations, are described in detail in^40^, from where the samples for the present study were derived. In brief, immunocompetent and syngenic adult Copenhagen rats (300–400 g, Charles River, Sulzfeld, Germany) were anesthetized with ketamine (75 mg/kg i.p.) and medetomidine (0.5 mg/kg i.p.), and an incision was made in the lower abdomen to expose the ventral prostate lobes. AT1 cells (ECACC, Sigma Aldrich catalogue number 94101449, cultured in RPMI 1640 + GlutaMAX medium supplemented with 10% fetal bovine serum (FBS) and 250 nM dexamethasone, 2 ×10^4^ cells in 10 µL RPMI 1640)) were injected into one of the ventral prostate lobes using a Hamilton syringe with a 30 G needle. The same procedure and culturing conditions were employed for MLL cells (ECACC, Sigma Aldrich catalogue number 94101454, 1 × 10^3^ cells). Ten days after the injections, the animals were anaesthetized and then killed by removal of the heart, and the prostate tissue was frozen in liquid nitrogen and stored at −80 °C. Prostate tissue from untreated rats (here termed HC) was also taken.

Ventral prostate tissues were microdissected on cryosections into tumour and TINT tissue, and the crysosections were then used for RNA extraction using the Allprep DNA/RNA/ Protein mini kit (Qiagen) (for details, see^40^). The microarray data reported in the present study, using an Affymetrix GeneTitan Gene 1.1 ST Rat array (Affymetrix), is taken from the S1 Dataset of^40^.

### IL-6 treatment of AT1 cells *in vitro*

AT1 cells (passage 18–41, obtained from the American Type Culture Collection, Manassas, VA, USA) were grown in 75cm^2^ cell culture bottles at 37 °C with 5% CO_2_ in humidified atmospheric pressure. The medium was RPMI 1640 supplemented with 4mM L-glutamine, 250 nM dexamethasone, 10% foetal bovine serum (FBS), 100 U/ml penicillin and 100 µg/ml streptomycin. The cells were passaged at confluence, approximately twice a week 1:8-1:10. For mRNA sample extraction, cells were plated in 12 well plates (4 × 10^5^ cells/well) in complete medium and allowed to settle. After 4–6 h of incubation, the medium was changed to serum-free medium. After an additional 16–18 h of incubation, the cells were washed with PBS, and then incubated for 3 h with modified Krebs-Ringer HEPES bicarbonate buffer (100 mM NaCl, 3.6 mm KCl, 0.5 mM NaH_2_PO_4_, 0.2 mM MgSO_4_, 1.5 mM CaCl_2_, 10 mM HEPES, 2 mM NaHCO_3_, pH 7.4) and rat recombinant IL-6 (0, 25 or 100 ng/ml final concentrations, in PBS supplemented with 0.1% BSA (final concentration 0.001%)). In the subsequent experiments, medium either with or without FBS (1%) was used in place of the modified Krebs-Ringer HEPES bicarbonate buffer, and the cells were incubated for 3 or 24 h, as indicated in the Legend to Fig. 5. The wells were washed once in cold PBS, and cells were collected followed by lysing in 300 µL of lysis/binding buffer (mRNA isolation, Thermo Fisher Scientific, Waltham, MA, USA) and storage at −80 °C.

### RT qPCR

For the *in vitro* experiments with AT1 cells, mRNA was isolated using Dynabeads mRNA Direct purification kit according to the manufacturer’s instructions. RNA concentration and purity was measured using a Nanodrop Lite spectrophotometer (Thermo Fisher Scientific). A 260/280 nm ratio ≥2 was considered acceptable, samples below 1 were discarded. RNA (50 ng) was converted to cDNA using the High-capacity cDNA Reverse Transcription Kit from Thermo-Fisher Scientific according to the protocol supplied by the manufacturer. Reverse transcription was performed in a Life Touch thermal cycler (Bioer, Hangzou, China), according to the following protocol: 25 °C × 10 min, 37 °C × 120 min, 85 °C × 5 min, thereafter 4 °C.

cDNA was diluted 10-fold in Tris-EDTA buffer (10 mM Tris, 0.1 mM EDTA pH 8.0) prior to use, except for *Psmc4, Cnr1, Cnr2, Mgll, Napepld* where undiluted cDNA was required to achieve acceptable melt curves. RT qPCR was performed using SYBR green as the reporter dye (qPCRBIO Sygreen Mix separate-ROX) in a an Illumina ECO Real-Time PCR system ECO Software v4.0.7.0 (Illumina Inc., San Diego, CA) according to the following protocol: 95 °C × 2 min followed by 45 cycles (95 °C × 10 s, 60 °C × 30 s) finishing with 95 °C × 15 s and a stepwise melt session between 55 °C and 95 °C. Samples (for low expressed mRNAs) were excluded when a pure one-product melt-curve could not be obtained. Data are presented as ∆Ct with respect to the reference gene (*Rpl19*) rather than 2^−∆∆Ct^ since the former allows for comparison of mRNA levels for the different genes. A difference of +1 or −1 between two groups represents a difference of 50% or 200%, respectively, of the mRNA levels. In the orthotopic experiments, two other reference genes were also used, *Rps12* and *Psmc4*. Thus, there are seven possible presentations of the data: the reference genes alone, the three pairwise combinations of the reference genes, and the mean for all three genes. We have presented the data with respect to *Rpl19*, but given the range of P values seen with the other six combinations in Tables 1 and 2.

### Hydrolysis of [^3^H]AEA

AT1 Cells were plated in 24 well plates (2 × 10^5^ cells/well) in complete medium and allowed to settle. After 4–6 h of incubation, the medium was changed to serum-free medium. After an additional 16–18 h of incubation, the cells were exposed to medium with or without FBS (1%) for 3 h at 37 °C. Thereafter, cells were washed once in modified Krebs-Ringer HEPES bicarbonate buffer supplemented with 1% BSA and then rinsed with the same buffer without BSA. Compounds (1 µM URB597, 30 µM pentadecylamine, the combination of both, or vehicle (0.01% DMSO, final concentration)) were added in the buffer supplemented with 0.1% fatty acid free BSA and the cells were preincubated with the compounds for 10 min at 37 °C before [^3^H]AEA (labelled in the ethanolamine part of the molecule and diluted with non-radioactive AEA to give a final concentration of 100 nM) was added. The hydrolysis reaction was allowed to proceed for 15 min at 37 °C before the reaction was stopped by addition of 600 µL charcoal: 0.5 M HCl (1:5)^50,69^. The samples were mixed and aliquots (600 µL) were pelleted by centrifugation 10 min at 1200 × *g*, room-temperature. Aliquots (200 μL) of the aqueous phase containing the [^3^H]ethanolamine hydrolysis product were transferred to scintillation vials together with scintillation fluid and analysed for tritium content by liquid scintillation with quench correction. Blanks were wells without cells. Additional parallel plates were included in each treatment session for measurement of protein content using Pierce BCA protein assay kits, and the data are expressed as pmol of [^3^H]AEA hydrolysed per mg protein.

### Statistics

Data are shown as means ± SD or graphically as scatterplots with means shown as bars. For the orthotopic data, one-way Welch’s ANOVAs, which do not assume equal SD values, were calculated using the statistical package built into GraphPad Prism v 8 for the Macintosh (GraphPad Software Inc., San Diego, CA, USA). For the data with cultured AT1 cells, we have followed the advice of Lew^70^ that a randomised block ANOVA (i.e. where both experiment day and treatment are included as factors) is more powerful than a standard one-way ANOVA when there is a day-to-day variation in the measured values. In consequence, the experimental data with the AT1 cells have been analysed either using mixed effects models (REML) not assuming sphericity (for the qPCR data, Fig. 5, GraphPad Prism) or for a three way mixed ANOVA with hc3 heteroscedascity correction (for the [^3^H]AEA hydrolysis experiments) using the function ezANOVA in the ez package v 4.4-0 for the R statistical programme v 3.6.0^71^. For the qPCR experiments, a critical value of P using a 5% false discovery rate^72^ was determined on Microsoft Excel spreadsheets.

## Supplementary information

Supplementary Information.

## Data Availability

The datasets generated during the current study are available from the corresponding author on reasonable request.
