# Supplementary material for: Effects of orthotopic implantation of rat prostate tumour cells upon components of the N-acylethanolamine and monoacylglycerol signalling systems: an mRNA study
Source: Sci Rep. 2020 Apr 14;10:6314. doi: 10.1038/s41598-020-63198-y (PMC7156441; doi:10.1038/s41598-020-63198-y)
Supplement: Supplementary file 1 — Supplementary Information. [file 41598_2020_63198_MOESM1_ESM.docx]

**Supplementary Information**

**Effects of orthotopic implantation of rat prostate tumour cells upon components of the *N*-acylethanolamine and monoacylglycerol signalling systems: an mRNA study**

**Mireille Alhouayek, Linda Stafberg, Jessica Karlsson, Sofia Halin Bergström and Christopher J. Fowler**

**Supplementary Table S1.** Primer pairs, target sequences and efficiencies.

|  |  | Primer pair | Eff. (%) | Target | Exon spanning |
| --- | --- | --- | --- | --- | --- |
| *Abhd6* | F:  R: | GACGTTCGCATCCCTCACAAC  CTGTGTTGGGACCTTGATCTTGTC | 90 | NM_001007680.1  (114: 771-884) | Yes |
| *Abhd12* | F:  R: | CATCTCGGCAGGAAGCTATAC  AGCCAAGGTCTGAGTGAAAG | 93 | NM_001024314.1  (94: 1173-1266) | Yes |
| *Cnr1* | F:  R: | GCTAGCTTCGGTTCGACATC  GGGAGAACCTGTATGAGGAGAG |  | NM_012784.5  (136: 3367-3502) | No |
| *Cnr2* | F:  R: | GTGCTACCCACCTACCTACA  TAGGAGATCAACGCCGAGAG | 103 | NM_001164143.3 (t.v.1)  (93: 675-767)  NM_001164142.3 (t.v.2)  (93: 573-665)  NM_020543.4 (t.v.3)  (93: 405-497) | No  No  No |
| *Dagla* | F:  R: | TTGTGACTGCTGTGGTTCTG  CCACGATGATCCGCCATTT | 99 | NM_001005886.1  (129: 1637-1765) | Yes |
| *Daglb* | F:  R: | TTGTGGTTGCCGTGAGAG  GCTTGAGCAATTCCCTTGTG | 91 | NM_001107120.1  (127: 1188-1314) | Yes |
| *Faah* | F:  R: | AAGGGCGGCGTTGATGTAAG  AATGATGGCAGGCGGAGTTC | 107 | NM_001369126.1  (81: 3315-3395) | No |
| *Mgll* | F:  R: | CGACTTTGAAGGTCCTTGCTG  AGATGAGTGGGTCGGAGTTG |  | NM_138502.2  (132:587-718) | Yes |
| *Naaa* | F:  R: | CCACTGAAGAAGGGAACAGAC  TCTGCTACTGAGCCTCACC | 101 | NM_001010967.2  (108: 1414-1521) | No |
| *Napepld* | F:  R: | ACTGGTTACTGCCCTGCTTT  AATCCTTACAGCATCCTCTGGG | 94 | NM_199381.1  (138:853-990) | Yes |
| *Psmc4* | F:  R: | CATCTGTCAGGAGAGTGGAATG  GCTTTCTCGAAGTCCTTGGC | 96 | NM_057122.1  (75:1143-1217) | Yes |
| *Ptgs2* | F:  R: | TGAGCGGTTACCACTTCAAA  TGCCAGTGATAGAGTGTGTTG | 91 | NM_017232.3  (109: 1013-1121) | No |
| *Rpl19* | F:  R: | TACTGCCAACGCTCGGAT  AACACATTCCCTTTGACCTTCA | 93 | NM_031103.1  (147: 277-423) | Yes |
| *Rps12* | F:  R: | GAGCACCAGATCAACCTGATAAAG  GCCATAGTCCTTAACTACAACGC | 91 | NM_031709.3  (135: 329-463) | Yes |
| *Il6r* | F:  R: | CCACACAGGTCTCTGTTGAAG  GAGGACACTCGTTGCTTCTG |  | NM_017020.3  (77: 999-1075) | Yes |
| *Tnfa* | F:  R: | GTGATCGGTCCCAACAAGG  GGTGGTTTGCTACGACGTG |  | NM_012675.3  (136: 307-442) | Yes |

Abbreviation: Eff., efficiency, when determined. Primer specificities were checked using Primer-BLAST at <https://www.ncbi.nlm.nih.gov/tools/primer-blast/>. Product lengths >700 base pairs are not shown in the Table. The numbers in brackets under the NM_ target information refer to the target sequence length and region to which the primer pair binds.

**Supplementary Table S2.** Comparison of the array data values for host control (HC, N=8), AT1 tumour tissue (N=8) and MLL tumour tissue (N=7).

|  |  |  | |  | |  | | ANOVA | Post hoc Dunnett’s T3 tests | | |
| --- | --- | --- | --- | --- | --- | --- | --- | --- | --- | --- | --- |
|  | Feature | HC | | AT1 tumour | | MLL tumour | | P value | HC vs | HC vs | AT1 vs |
|  | ID | mean | SD | mean | SD | mean | SD | (Welch) | AT1 | MLL | MLL |
| ***Anabolic*** |  |  |  |  |  |  |  |  |  |  |  |
| *Napepld* | 10853132 | 5.97 | 0.16 | 6.23 | 0.11 | 7.97 | 0.21 | <10^-9^ | 0.0080 | 1.0x10^-9^ | 3.0x10^-8^ |
| *Dagla* | 10728676 | 5.55 | 0.15 | 7.83 | 0.34 | 7.61 | 0.14 | <10^-9^ | 2.6x10^-8^ | <10^-9^ | 0.32 |
| *Daglb* | 10760403 | 7.42 | 0.08 | 8.56 | 0.16 | 8.05 | 0.13 | 2x10^-9^ | 2.3x10^-8^ | 2.1x10^-6^ | 4.3x10^-5^ |
|  |  |  |  |  |  |  |  |  |  |  |  |
| ***Target*** |  |  |  |  |  |  |  |  |  |  |  |
| *Cnr1* | 10868186 | 5.05 | 0.18 | 3.76 | 0.27 | 3.63 | 0.09 | <10^-9^ | 2.6x10^-7^ | 6.0x10^-9^ | 0.47 |
| *Cnr2* | 10872935 | 5.86 | 0.10 | 7.44 | 0.19 | 6.58 | 0.21 | 1x10^-9^ | 1.0x10^-9^ | 9.7x10^-5^ | 9.8x10^-6^ |
|  |  |  |  |  |  |  |  |  |  |  |  |
| ***Catabolic*** |  |  |  |  |  |  |  |  |  |  |  |
| *Faah* | 10871182 | 8.34 | 0.10 | 5.82 | 0.53 | 6.05 | 0.22 | <10^-9^ | 3.1x10^-6^ | 2.1x10^-8^ | 0.62 |
| *Naaa* | 10771690 | 6.75 | 0.21 | 9.35 | 0.37 | 7.82 | 0.32 | 3x10^-9^ | 7.0x10^-9^ | 6.0x10^-5^ | 2.9x10^-6^ |
| *Mgll* | 10857130 | 7.35 | 0.19 | 6.71 | 0.23 | 8.05 | 0.16 | 2.5x10^-8^ | 9.9x10^-5^ | 1.1x10^-5^ | 4.9x10^-8^ |
| *Abhd6* | 10782695 | 10.36 | 0.04 | 10.12 | 0.10 | 9.27 | 0.12 | 2x10^-9^ | 5.2x10^-4^ | 2.3x10^-7^ | 1.6x10^-8^ |
| *Abhd12* | 10850668 | 10.30 | 0.09 | 10.15 | 0.08 | 9.35 | 0.10 | <10^-9^ | 0.010 | <10^-9^ | 2.0x10^-9^ |
| *Ptgs2* | 10764551 | 5.39 | 0.31 | 6.56 | 0.44 | 6.31 | 0.78 | 2.1x10^-4^ | 1.1x10^-4^ | 0.053 | 0.84 |
|  |  |  |  |  |  |  |  |  |  |  |  |
| ***Other*** |  |  |  |  |  |  |  |  |  |  |  |
| *Tnfa* | 10828021 | 5.22 | 0.29 | 7.09 | 0.49 | 5.98 | 0.41 | 3.2x10^-6^ | 4.8x10^-6^ | 0.0050 | 0.0011 |

The data are taken from the dataset given as S1 Dataset in Strömvall *et al.* (ref ^40^ in main article) and are normalised values on a log_2_ scale. Note that the dataset returns three sets of values for *Abhd12*: ID 10850668, recognizing NM_001024314 (shown in the Table); ID10850682, recognizing the putative gene BC078918; and 10884996, recognizing the gene NM_001003928 (*Abhd12b*). The HC control mean values for the latter two (5.31 and 3.08) were much lower than for the *Abhd12* data shown in the table. For the ANOVA P values, which were calculated not assuming equal SD values, the critical value of P assuming a 5% false discovery rate^72^ was 0.05, although it should be taken into account that these data are part of a much larger dataset.^40^

**Supplementary Table S3.** Comparison of the array data values for host control (HC, N=8), AT1 TINT (N=6) and MLL TINT (N=7).

|  |  |  | |  | |  | | ANOVA | Post hoc Dunnett’s T3 tests | | |
| --- | --- | --- | --- | --- | --- | --- | --- | --- | --- | --- | --- |
|  | Feature | HC | | AT1 TINT | | MLL TINT | | P value | HC vs | HC vs | AT1 vs |
|  | ID | mean | SD | mean | SD | mean | SD | (Welch) | AT1 | MLL | MLL |
| ***Anabolic*** |  |  |  |  |  |  |  |  |  |  |  |
| *Napepld* | 10853132 | 5.97 | 0.16 | 5.86 | 0.13 | 5.96 | 0.15 | 0.29 | 0.38 | 1.00 | 0.45 |
| *Dagla* | 10728676 | 5.55 | 0.15 | 5.47 | 0.09 | 5.53 | 0.10 | 0.38 | 0.51 | 0.98 | 0.58 |
| *Daglb* | 10760403 | 7.42 | 0.08 | 7.40 | 0.09 | 7.46 | 0.07 | 0.35 | 0.98 | 0.59 | 0.44 |
|  |  |  |  |  |  |  |  |  |  |  |  |
| ***Target*** |  |  |  |  |  |  |  |  |  |  |  |
| *Cnr1* | 10868186 | 5.05 | 0.18 | 4.58 | 0.18 | 4.76 | 0.19 | 0.0018 | 0.0016 | 0.028 | 0.27 |
| *Cnr2* | 10872935 | 5.86 | 0.10 | 5.81 | 0.21 | 5.84 | 0.20 | 0.90 | 0.95 | 1.00 | 0.99 |
|  |  |  |  |  |  |  |  |  |  |  |  |
| ***Catabolic*** |  |  |  |  |  |  |  |  |  |  |  |
| *Faah* | 10871182 | 8.34 | 0.10 | 8.33 | 0.19 | 8.40 | 0.18 | 0.72 | 1.00 | 0.80 | 0.87 |
| *Naaa* | 10771690 | 6.75 | 0.21 | 6.87 | 0.41 | 7.05 | 0.49 | 0.36 | 0.88 | 0.41 | 0.86 |
| *Mgll* | 10857130 | 7.35 | 0.19 | 7.18 | 0.23 | 7.18 | 0.22 | 0.26 | 0.42 | 0.37 | 1.00 |
| *Abhd6* | 10782695 | 10.36 | 0.04 | 10.44 | 0.14 | 10.28 | 0.18 | 0.27 | 0.46 | 0.66 | 0.26 |
| *Abhd12* | 10850668 | 10.30 | 0.09 | 10.25 | 0.06 | 10.45 | 0.10 | 0.0034 | 0.51 | 0.024 | 0.0034 |
| *Ptgs2* | 10764551 | 5.39 | 0.31 | 5.40 | 0.21 | 5.66 | 0.41 | 0.36 | 1.00 | 0.45 | 0.43 |
|  |  |  |  |  |  |  |  |  |  |  |  |
| ***Other*** |  |  |  |  |  |  |  |  |  |  |  |
| *Tnfa* | 10828021 | 5.22 | 0.29 | 5.11 | 0.28 | 5.23 | 0.38 | 0.75 | 0.86 | 1.00 | 0.88 |

The data are taken from the dataset given as S1 Dataset in Strömvall *et al.* (ref ^40^ in main article) and are normalised values on a log_2_ scale. See Legend to Supplementary Table S2 regarding the values for *Abhd12*. Note that the HC values are the same as in Supplementary Table S2. For the ANOVA P values, which were calculated not assuming equal SD values, the critical value of P assuming a 5% false discovery rate was 0.0083, although it should be taken into account that these data are part of a much larger dataset.^40^

**Supplementary Fig. S1.** Scatterplots of *Dagla, Daglb,* *Cnr1*, *Faah, Abhd6, Abhd12* and *Tnfa* gene expression in host control (HC), tumour (tu) tissue and TINT. Comparison of qPCR and Array data.

Left axes show the ∆Ct from the qPCR experiments with *Rpl19* as reference gene. The right axes show the array data, as normalised values on a log_2_ scale, taken from S1 Dataset in Strömvall *et al.*^40^ The statistical analyses of these data are presented in Tables 1 and 2 (qPCR) of the paper and in Supplementary Tables S2 and S3 (array). Note that the left y-axis but not the right y-axis has been reversed. Thus for both qPCR and Arrays, the direction of change (-1 corresponds to a doubling for the qPCR data, while +1 corresponds to a doubling for the array data) is the same.

**Supplementary Fig. S2.**  Volcano plots of bivariate comparisons between a. host control (“HC”) and tumour tissue; b. HC and TINT. Array data.

The log_2_ fold change is calculated from the mean normalised values summarized in Supplementary Tables 2 and 3. Note the different scales in Panels a and b. The P values are for the post-hoc comparisons given in these Tables. The vertical dotted lines show a fold change of ±1, i.e. a halving/doubling of mRNA expression. The horizontal lines show the critical value of P assuming a 5% false discovery rate (0.043 for Panel a, 0.0014 for Panel b). The genes are numbered as follows: 1, *Napepld*; 2, *Dagla*; 3, *Daglb*; 4, *Cnr1*; 5, *Cnr2*; 6, *Faah*; 7, *Naaa*; 8, *Mgll*; 9, *Abhd6*; 10, *Abhd12*; 11, *Ptgs2* and 12, *Tnfa*.

**Supplementary Fig. S3.** Comparison of the mRNA levels seen in cultured AT1 cells and host control (HC) prostate tissue.

The abscissae show the ∆Ct values for the cultured cells (the same data as shown in Fig. 4A) and the ordinates show the corresponding values (summarized in Table 1) for the HC tissue. Each point represents the mean ± SD. The blue dotted line is the line of identity. The grey lines show where the HC expression is 10-fold higher (upper line) or lower (lower line) than the expression in the AT1 cells. It should be mentioned as a caveat that the experiments were undertaken on different occasions. The genes are numbered as follows: 1, *Napepld*; 2, *Dagla*; 3, *Daglb*; 4, *Cnr1*; 5, *Cnr2*; 6, *Faah*; 7, *Naaa*; 8, *Mgll*; 9, *Abhd6*; 10, *Abhd12* and 11, *Ptgs2*.
